# An open pilot study of zonisamide augmentation in major depressive patients not responding to a low dose trial with duloxetine: preliminary results on tolerability and clinical effects

**DOI:** 10.1186/1744-859X-10-23

**Published:** 2011-09-19

**Authors:** Michele Fornaro, Matteo Martino, Bruna Dalmasso, Salvatore Colicchio, Marzia Benvenuti, Giulio Rocchi, Andrea Escelsior, Giulio Perugi

**Affiliations:** 1 Department of Neuroscience, Section of Psychiatry, University of Genova, Genoa, Italy; 2 Department of Hematology and Oncology, Section of Semeiotics and Medical Methodology I, University of Genova, Genoa, Italy; 3 Department of Neurosciences, Catholic University of Rome, Rome, Italy; 4 Department of Psychiatry, University of Pisa, Pisa, Italy

## Abstract

**Background:**

Despite multiple antidepressant options, major depressive disorder (MDD) still faces high non-response rates, eventually requiring anticonvulsant augmentation strategies too. The aim of this study was to explore such a potential role for zonisamide.

**Methods:**

A total of 40 MDD outpatients diagnosed using the *Diagnostic and Statistical Manual for Mental Disorders*, fourth edition criteria entered a 24 week open trial receiving duloxetine 60 mg/day for the first 12 weeks and subsequently (weeks 12 to 24) augmentation with zonisamide 75 mg/day if they did not respond to the initial monotherapy. Efficacy and tolerability were assessed using the Hamilton Scales for Anxiety and Depression (a 12 week score ≥50% vs baseline defined 'non-response'), the Arizona Sexual Experience Scale, the Patient Rated Inventory of Side Effects and the Young Mania Rating Scale.

**Results:**

At week 12, 15 patients out of 39 (38.5%) were responders, and 1 had dropped out; remarkably, 14 patients out of 24 (58.3%) had achieved response by week 24. Poor concentration and general malaise were associated with non-response both at week 12 and 24 (P = 0.001), while loss of libido and reduced energy were prominent among final timepoint non-responders. Patients receiving zonisamide also experienced weight reduction (2.09 ± 12.14 kg; P = 0.001) independently of the outcome.

**Conclusions:**

Although only a preliminary study due to strong methodological limitations, and thus requiring confirmation by further controlled investigations, the current results indicate zonisamide may be a potential augmentation option for some depressed patients receiving low doses of duloxetine.

## Introduction

Major depressive disorder (MDD) is significant cause of morbidity and mortality, and is associated with a high socioeconomic burden [1]. Both pharmacological and non-pharmacological interventions have proven efficacy in many MDD cases, yet failure to respond to standard interventions still represents a frequent scenario [2]. Among other issues, nosological boundaries and pharmacological issues might lead to unfavorable outcomes, prompting pharmacological augmentation strategies [3] even for 'proven effective' antidepressants such as the serotonergic norepinephrinergic reuptake inhibitors (SNRIs) [4].

A pharmacological augmentation strategy involves a number of agents from different classes, including some anticonvulsants routinely prescribed in the clinical setting both for anxious states [5] and/or depression [6], sometimes controversially [7,8] or even in the absence of a bipolar course of illness [6].

To mention one, lamotrigine is an anticonvulsant drug often used as an antidepressant augmentation therapy even for non-bipolar patients, although its use as augmentation strategy for treatment-resistant unipolar depression is supported by only a single randomized clinical trial [9]. While it may be argued that many *Diagnostic and Statistical Manual for Mental Disorders*, fourth edition (DSM-IV) [10] cases of depression should indeed follow a bipolar diathesis, suggesting prudent (or low dose) prescription of antidepressants and/or augmentation therapies with antimanic agents [2], such a prescribing habit is a popular clinical practice also supported by pharmacodynamic considerations. With regard to lamotrigine, its actions include blockade of sodium and calcium channels, hypothetically leading to reduced *N*-methyl-D-aspartate glutamatergic transmission as well as changes in the activity of crucial neurotransmitters involved in the pathophysiology of depression, including dopamine and serotonin [11-13]. Therefore, in consideration of a partial pharmacodynamic overlap between lamotrigine and the latterly introduced zonisamide [14-16] (at least with regard to a common putative modulation of glutamate and monoamines), and accounting for the drug concentration-related biphasic effects of zonisamide on serotonergic system functioning in rat hippocampus [15], zonisamide should also receive attention for its potential role in the management of some psychiatric disturbances, as recently proposed for anxiety disorders refractory to standard anxiolytic medications [17]. Additionally, zonisamide (a sulfonate anticonvulsant drug with long half life (65 h in plasma) approved for use as an adjunctive therapy in adults with partial-onset seizures, infantile spasm, mixed seizure types of Lennox-Gastaut syndrome, myoclonic, and generalized tonic clonic seizure), when added at 25 to 50 mg/day to commonly used anti-Parkinsonian drugs, significantly improved the primary symptoms of patients with advanced Parkinson's disease, possibly by activation of dopamine synthesis, inhibition of monoamine oxidase type B, inhibition of T-type calcium channels and inhibition of an indirect pathway in the basal ganglia through the sigma opioid receptor [18]. Therefore, zonisamide's propensity to facilitate dopaminergic and serotonergic release *in vivo *[19] might suggest an exploration of its potential role as augmentation strategy for common antidepressant drugs is prudent, possibly even at dosages lower than the ones usually adopted for the treatment of epileptic conditions.

Therefore, in this study we explore the efficacy and tolerability of adjunctive zonisamide in the treatment of MDD not responsive to a preliminary trial of the SNRI antidepressant duloxetine administered at low dose (60 mg/day).

## Methods

### Study design

This was a preliminary 24 week open trial designed to assess the efficacy and tolerability of zonisamide 75 mg/day augmentation for MDD patients (actually an off-label prescription of zonisamide) not responding to a 12 week treatment with duloxetine at 60 mg/day. The unusual choice of duloxetine was essentially dictated by pharmacokinetic and pharmacodynamic issues, in view of subsequent combination with zonisamide. The study was conducted from February 2008 to September 2010, with approval by the Ethical Committee of the San Martino Hospital, University of Genova, Genoa, Italy in November 2007.

### Subjects

The planned and actual study population included 40 outpatients, aged 18 years or older, of both genders, fulfilling DSM-IV criteria for MDD and with a current single or recurrent major depressive episode. At screening, patients had to have a minimum score of 18 on the 17-item Hamilton Scale for Depression (HAM-D) [20]. Exclusion criteria included the following DSM-IV-defined diagnoses: bipolar disorders (either type I or type II), cyclothymia, schizoaffective disorder or schizophrenia, dementia or substance abuse disorder in the last 6 months, suicidal ideation that made participation unduly risky, unstable medical conditions, abnormal thyroid function, QTc ≥450 ms on screening electrocardiogram (ECG; calculated using the Bazett formula), being pregnant, lactating, or not using adequate contraception if capable of getting pregnant, as well as having known contraindications for zonisamide (for example, history of severe myopia, kidney stones or narrow angle glaucoma) or duloxetine. Patients were also excluded if unwilling or unable to provide valid signed informed consent or if they could not safely taper concomitant psychotropic drugs, which had to be withdrawn for 2 weeks, except for fluoxetine and depot neuroleptics requiring at least a 4 week discontinuation. Zolpidem 10 mg at bedtime was allowed, but could not be taken the night before scheduled assessments. Finally, body weight (in kg) was also recorded at screening, week 12 (main evaluation time) and week 24 (endpoint) along with repeated medical monitoring including ECG recording. Patients could leave the study at any time and still obtain clinical care.

### Study procedures and efficacy measures

Diagnoses were made by clinical examination and the Structured Clinical Interview for DSM-IV Axis-I Disorders/Patient Edition (SCID-I/P) [21]. At baseline, eligible patients started taking duloxetine 60 mg/day once a day after being evaluated by means of the HAM-D, Hamilton Scale for Anxiety (HAM-A) [22], Young Mania Rating Scale (YMRS) [23] and the Arizona Sexual Experience Scale (ASEX) [24]. All measurements were repeated at week 12 and week 24 while the Patient Rated Inventory of Side Effects (PRISE) [25] was administered at week 12 and week 24 as primary tolerability evaluation. As major outcome measurement, a week 12 HAM-D total score ≤50% vs baseline defined 'non-response'. Similarly, an endpoint HAM-D total score ≤50% vs baseline was adopted to define (final) 'responders' (primary endpoint) or 'remission' if < 7.

### Data analysis

Both descriptive and analytical analyses (χ^2 ^or t tests when appropriate) were performed using SPSS V.19 for Windows (SPSS, Chicago, IL, USA). Two-tailed tests with a 5% level of significance were used through the analyses. Since the data followed a normal distribution (assessed by Kolmogorov-Smirnov test), only parametric analyses were conducted. Finally, as a result of the very low number of dropout cases (in fact, n = 1; see below) an intent to treat analysis was performed.

## Results

**Figure 1:**
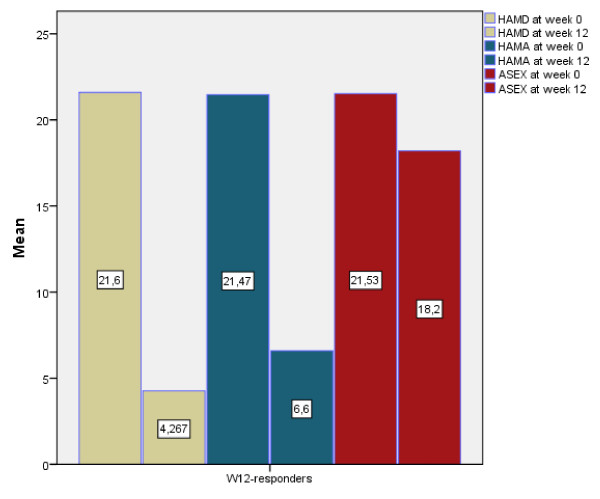
**Mean values for HAM-D, HAM-A and ASEX at baseline and week 12 in responder patients concluding the trial prior to receiving zonisamide augmentation**. ASEX = Arizona Sexual Experience Scale; HAM-A = Hamilton Anxiety Scale; HAM-D = Hamilton Depression Scale; YMRS = Young Mania Rating Scale.

A total of 40 patients, all of Caucasian origin, constituted the study sample. Two patients dropped out before week 12 and week 24, respectively. The first dropout case did not attend the scheduled follow-up, giving no reason, while the second did not complete the final follow-up for (clinically confirmed) depressed mood: although not fulfilling the HAM-D scale at week 24, this subject was included in the 'non-responders' group, being therefore considered in the final statistical analysis. The mean HAM-D reduction for the group as a whole from baseline (20.53) to week 12 (10.08) was -10.45. At screening, mean age was (47 ± 10.7), F = 24 (60%) and M = 16 (40%); none of the patients had relevant medical or psychiatric Axis-I comorbidities and 84% of the sample experienced first (considered as single) major depressive episode, with a mean baseline HAM-D score = 23 (18 to 24 scores indicate moderate/average depression). At week 12, 15 out of 39 (38.5%) subjects were 'responders' while 24 (61%) were not, thus continuing the study with zonisamide 75 mg/day once a day augmentation and maintaining duloxetine 60 mg/day once a day. By definition, week 12 HAM-D total scores were significantly lower among responders (*P *= 0.001) as this was the case of HAM-A total scores (*P *= 0.001), Figure 1.

**Figure 2:**
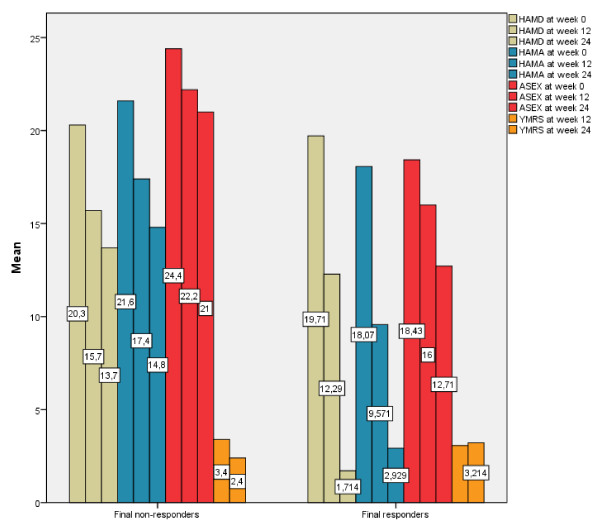
**Trends of HAM-D, HAM-A, ASEX and YMRS from baseline to week 12 and to week 24 in final responders and final non-responders**. ASEX = Arizona Sexual Experience Scale; HAM-A = Hamilton Anxiety Scale; HAM-D = Hamilton Depression Scale; YMRS = Young Mania Rating Scale.

**Figure 3:**
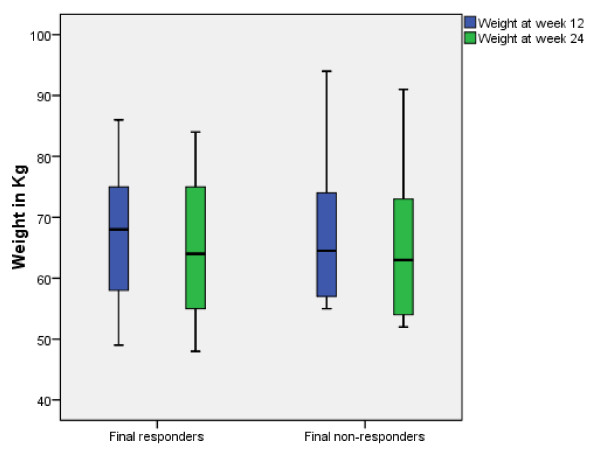
**Comparison of mean weight (in kg) at weeks 12 and 24 in final responders vs final responders**. Those patients receiving zonisamide experienced a slight, yet statistically significant (*P *= 0.001 both) weight reduction independently of the outcome. ASEX = Arizona Sexual Experience Scale; HAM-A = Hamilton Anxiety Scale; HAM-D = Hamilton Depression Scale; YMRS = Young Mania Rating Scale.

With regard to the side effects profile, 'anorgasmy' (*P *= 0.003), 'poor concentration' and 'general malaise' (both *P *= 0.001) were more frequent among preliminary non-responders vs responders, as shown in Table 1. At week 24, the general sexual (side effect) profile (ASEX total) was poorer in final non-responders (n = 10, 41.7%), with 'loss of libido' and 'anorgasmy' (both *P *= 0.001) among the most frequent complaints; 'general malaise' and 'poor concentration' remained prevalent among non-responders (both *P *= 0.001), associated with 'reduced energy' (*P *= 0.001) compared to final responders (n = 14, 58.3%), as reported in Figure 2 and Table 2. No patients evolved to a manic episode (defined by YMRS total score ≥13) or developed clinically relevant medical adverse events during the follow-up period. Remarkably, patients treated with zonisamide experienced significant weight reduction (mean 2.09 ± 12.14 kg; *P *= 0.001) independently of their final outcome (mean 2.79 ± 11.67 kg and 1.39 ± 12.61 kg in responders and non-responders, respectively), as shown in Figure 3, whereas mean week 12 weight did not statistically differ from baseline values.

**Table 1:** Demographic and clinical features week 12 comparison of responders vs non-responders

	Responders, N = 15 (38.5%)	Non-responders, N = 24 (61.5%)	**t or χ**^**2**^**(df = 1)**	*P *value
Age, mean(s)	41.33 ± 9.5	50.7 ± 0.2	2.865	NS

General features	F = 9; M = 6	F = 1; M = 23	-0.152	NS

Weight (in kg)	68.4 ± 15.71	68.29 ± 12.11	36	NS

Clinical features (%)				

HAM-D total score	4.27 ± 3.15	13.71 ± 2.53	10.320	0.001

HAM-A total score	6.60 ± 3.58	12.83 ± 4.76	4.351	0.001

YMRS total score	1.93 ± 1.62	3.21 ± 1.16	2.394	0.022

ASEX total score	18.20 ± 5.69	18.58 ± 4.87	0.224	NS

Side effects profile (%)				

Diarrhea	0	4 (10.25%)	2.786	NS

Tremors	0	2 (5.12%)	1.318	NS

Poor coordination	0	2 (5.12%)	1.318	NS

Dizziness	0	4 (10.25%)	2.786	NS

Blurred vision	3 (7.7%)	6 (15.4%)	0.130	NS

Ringing in ears	4 (10.25%)	0	7.131	0.008

Difficult urination	4 (10.25%)	2 (5.12%)	2.383	NS

Painful urination	0	2 (5.12%)	1.318	NS

Pollachiuria	1 (2.6%)	2 (5.12%)	0.036	NS

Menstrual irregularity	0	0	-	-

Insomnia	11 (28.2%)	18 (46.15%)	0.013	NS

Hypersomnia	0	4 (10.25%)	2.786	NS

Loss of libido	4 (10.25%)	12 (30.77%)	2.077	NS

Anorgasmy	4 (10.25%)	18 (46.15%)	8.770	0.003

Erectile dysfunction	0	0	-	-

Poor concentration	1 (2.6%)	16 (41%)	13.514	0.001

General malaise	0	14 (35.9%)	13.650	0.001

Reduced energy	3 (7.7%)	12 (30.77%)	3.510	NS

Hyperphagia	2 (5.12%)	4 (10.25%)	0.079	NS

ASEX = Arizona Sexual Experience Scale; HAM-A = Hamilton Anxiety Scale; HAM-D = Hamilton Depression Scale; NS = not significant; YMRS = Young Mania Rating Scale.

**Table 2:** Week 24 comparison of demographic and clinical features of responders vs non-responders

	Responders, N = 14 (58.3%)	Non-responders, N = 10 (41.7%)	**t or χ**^**2**^**(df = 1)**	*P *value
Weight (in kg)	65.5 ± 11.23	65.90 ± 13.12	22	NS

Clinical features (%)				

HAM-D total score	1.71 ± 1.32	13.70 ± 5.14	8.405	0.001

HAM-A total score	2.93 ± 2.05	14.80 ± 4.21	9.182	0.001

YMRS total score	3.21 ± 5.76	2.40 ± .843	-0.440	NS

ASEX total score	12.71 ± 1.89	18.58 ± 4.87	5.129	0.001

Side effects profile (%)				

Diarrhea	0	0	-	-

Tremors	0	0	-	-

Poor coordination	0	2 (8.3%)	3.055	NS

Dizziness	0	0	-	-

Blurred vision	0	0	-	-

Ringing in ears	0	0	-	-

Difficult urination	0	4 (16.7%)	6.720	0.010

Painful urination	0	0	-	-

Pollachiuria	0	4 (16.7%)	6.720	0.010

Menstrual irregularity	0	0	-	-

Insomnia	2 (8.3%)	4 (16.7%)	2.057	NS

Hypersomnia	0	2 (8.3%)	3.055	NS

Loss of libido	0	8 (33.3%)	16.800	0.001

Anorgasmy	0	6 (25%)	11.200	0.001

Erectile dysfunction	0	0	-	-

Poor concentration	0	10 (41.7%)	24.000	0.001

General malaise	0	6 (25%)	11.200	0.001

Reduced energy	0	10 (41.7%)	24.000	0.001

Hyperphagia	0	0	-	-

'Anorgasmy', 'poor concentration' and 'general malaise' were already significantly improved in those patients responding to a 12 week trial with duloxetine monotherapy and appear to still be associated to response even at week 24 among those patients who continued the study. Also, the difference in the ASEX scores between responders and non-responders at the end of the study is likely due to the fact that the non-responders' scores substantially did not modify within week 12 (before zonisamide augmentation) and week 24, while it reduced in final responders.

ASEX = Arizona Sexual Experience Scale; HAM-A = Hamilton Anxiety Scale; HAM-D = Hamilton Depression Scale; NS = not significant; YMRS = Young Mania Rating Scale.

## Discussion

By the end of the study, 29 (72.5%) out 40 patients had achieved response (51.7% with duloxetine monotherapy and 48.3% with both duloxetine and zonisamide). Nonetheless, a number of issues must be raised prior to considering the findings from the present pilot study. Primarily, this was a small sample, low-powered, open trial, thus its validity is limited by the absence of a control (and regression analysis techniques). Also, while the sample appeared quite homogeneous and prone to good compliance toward medications, it included mainly first (possibly single) episode major depressed patients with mainly mild to moderate cases of depression (as indicated by respective baseline HAM-D scores), thus making the study prone to a Berkson bias ('exclusion of most severe cases leading to potential distortion of statistical results'). Moreover, stating the explorative nature of this pilot study, we used low doses of drugs essentially for safety considerations. Hypothetically, some of the patients not responding at week 12 on duloxetine fixed-dose monotherapy may have responded if treated with higher doses of antidepressant (for example, 120 mg/day) and/or if treated for longer, although when an antidepressant response is observed it usually begins within the first months of treatment [26]. They may have also responded to an eventual placebo or even spontaneously due to the natural course of MDD. In this sense, it cannot be determined if and how any of the patients receiving zonisamide represented a true 'treatment resistant depression' case.

While this remains a major constraint of this pilot study, the use of low dosages of zonisamide (compared to anticonvulsant ranges) was essentially due to the explorative nature of the investigation and absence of specific guidance for its use for MDD. However, it should be considered that zonisamide is commonly used at dosages between 25 to 50 mg/day as augmentation therapy for common anti-Parkinsonian drugs. Moreover, while the concomitant use of duloxetine and zonisamide for the last 12 weeks of the study should be seen as a further confounding factor in discriminating the therapeutic effect of each single agent, this pilot study rather aimed to investigate the role of the combination of the two drugs, and the unusual choice of the SNRI duloxetine (mainly metabolized by CYP1A2 and CYP2D6) was essentially due to its low propensity for pharmacokinetic interactions with zonisamide (mainly metabolized via CYP3A4).

Finally, the present findings and hypotheses must be considered as merely speculative due to the shortcomings listed above. The present investigation was a small open-label uncontrolled study, therefore no firm conclusions can be drawn from the results; it should be considered as a pilot study for further rigorous investigation, essentially prompted by the fact that zonisamide appeared to be a well tolerated augmentation therapy (however, the absence of placebo control might be somehow misleading), with low dropout rates even in the presence of some side effects (although it should be remembered that people who recover from depression, for whatever reason, are also less likely to endorse a list of somatic complaints and that some other complaints, including sexual ones, could be part of MDD rather than side effects due to treatment). What should be noted is that zonisamide apparently did not produce negative effects compared with the start of treatment and that, since weight gain is a common complaint among MDD patients receiving standard antidepressants and a major potential cause of drug withdrawal, the observation of weight reduction in the presence of zonisamide augmentation added to a low-dose duloxetine suggests further methodologically rigorous, controlled studies would be warranted.

## Competing interests

The authors declare that they have no competing interests, including any connection to Eisai or Elan.

## Authors' contributions

MF conceived the study and performed the statistical analysis, MM contributed to manuscript drafting and BD performed the physical examinations. MB, GR, AE and SC helped in retrieving literature references and/or in patient enrollment and follow-up. GP served as senior study consultant. All authors read and approved the final version of the manuscript.
